# Ten maxims for out of class learning to outclass the academic challenges of COVID-19

**DOI:** 10.15694/mep.2020.000089.1

**Published:** 2020-05-05

**Authors:** Prashanti Eachempati, Komattil Ramnarayan

**Affiliations:** 1 Melaka-Manipal Medical College (Melaka Campus); 2 Melaka-Manipal Medical College

**Keywords:** Ten maxims, Online learning, Learning during pandemic, Out of class learning, Outclass COVID-19

## Abstract

This article was migrated. The article was marked as recommended.

Responding to the COVID-19 pandemic, universities world over are aggressively moving their learning, teaching and assessment to online platforms. Educators suddenly feel unsettled, unprepared and unsupported.

This inevitable segue from a traditional to online environment will need faculty to grapple quickly and efficiently with the technological imperatives. The purpose of this article is to present ten maxims to navigate the academic challenges posed by out of class learning. The article includes a user-friendly checklist to facilitate the blooming of online teachers.

## Introduction

**Figure 1.**
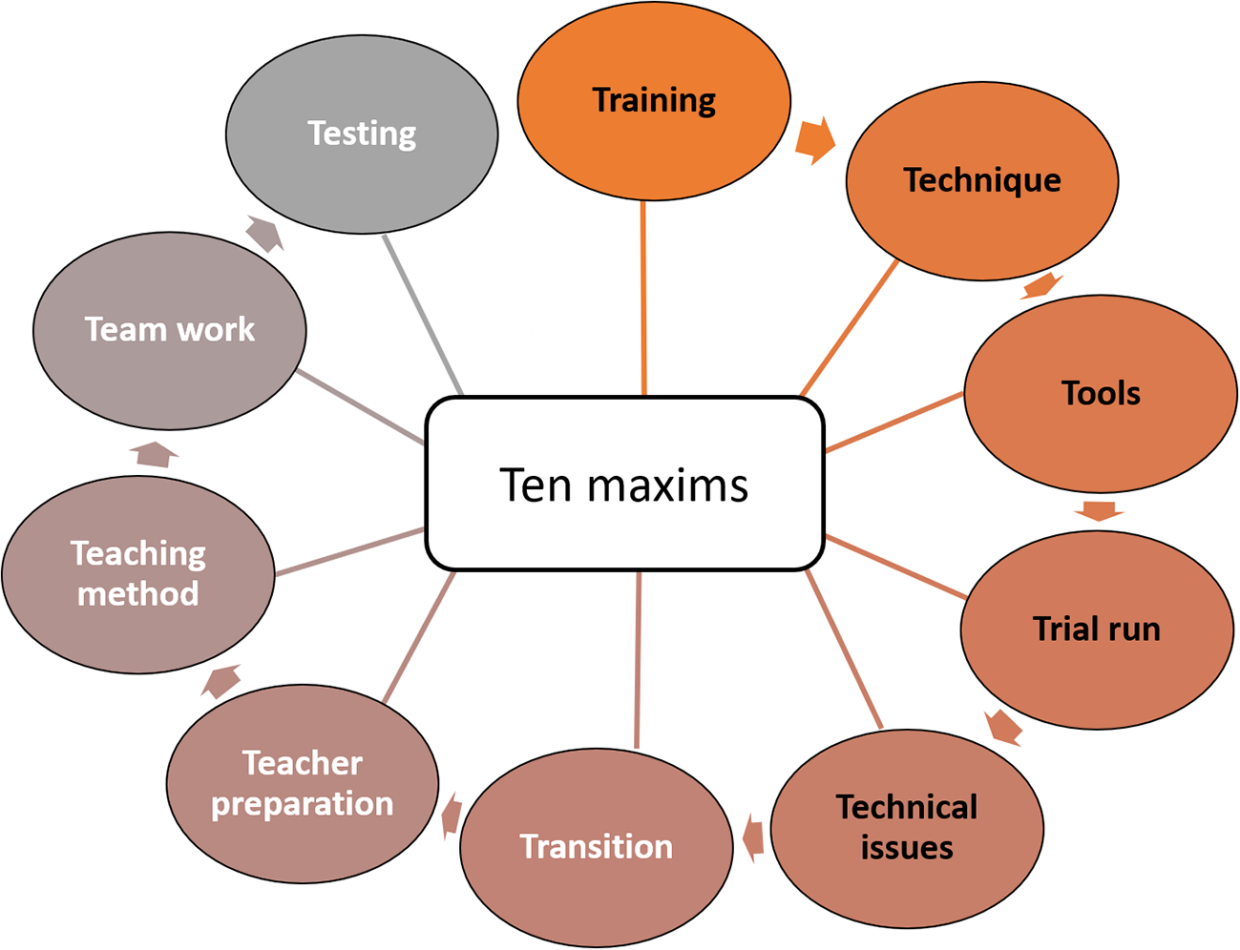
Ten maxims presented as ten T’s

In the aftermath of the World Health Organization’s declaration of the novel coronavirus as a pandemic, universities across the world have stopped face-face learning sessions in an attempt to slow its spread. All in-person classes are cancelled and there is a sudden shift of education to the virtual platform. However, the transition from traditional to online learning is not without challenges. This ‘imposed’ online learning has its issues. Although the current generation of students are technophiles and most comfortable with technology, the same may not be true with all the educators who belong to the previous generations. Research on the instructional uses of technology has revealed that teachers often lack the expertise to successfully integrate technology in their teaching and their attempts are wanting (or lacking) in scope, variety, and depth ( Mccormick and Scrimshaw, 2001). Hence, we present ten maxims to outlast the COVID-19 academic challenges using out of class learning. The maxims are presented using the 10 T’s ( Figure 1) which is to enable even the technophobic health professionals to seamlessly adapt to this pandemic crisis.

## Maxim 1: Training

### Technology enabled learning needs awareness

Instructing online requires a different skill set and instructors need to be alert to this need and aware of its potential ( Frass, Rucker and Washington, 2017). Success in distance education begins with orienting the faculty to its unique requirements. The current scenario of development of online instructors is variable at the individual and institutional level ( Frass, Rucker and Washington, 2017). There is a complex interplay of factors which govern the implementation of online learning - the interconnectedness between talk, technology and text must be carefully titrated ( Ching, Hsu and Rice, 2015).

With the constraints imposed by the pandemic, administrators must arrange to train all faculty without assuming that faculty will be able to do it without assistance. Prior to embarking on a training, it will be worthwhile conducting an internal needs assessment and current preparedness. While doing this, the principles of adult learning should be borne in mind ( McQuiggan, 2012). The information technology (IT) personnel should be an integral part of the team from the planning, implementation and evaluation stage. Institutions can look into creating a common platform for conducting online sessions which will help standardize the instructional strategies. IT team can assist in creating email id’s and password for all faculty and students so that they can log into a common platform. Classes can be conducted uniformly using this platform so that it is convenient for students and faculty alike. Stepwise instructions/ checklists to use the online tools, videos/tutorials which are simple to understand need to be shared with faculty. Arranging small group online discussions and one-one query clarifying sessions with IT personnel will be beneficial.

## Maxim 2: Technique

### Attempt a blended method of online learning

The key determinant that forcefully impacts online learning is the selection of the technique to deliver the course content. It is important for instructors to first determine whether they plan to engage students in online or distance education that happens in real-time, (synchronous) or an asynchronous manner where learning occurs through online channels without real-time interaction. Both the techniques have their merits and demerits ( Huang and Hsiao, 2012).

Allowing every student to pace his/her learning commensurate with his/her ability and need will instill confidence in and responsibility on the learner. This means that everyone is allowed to decide how, when, and where to learn. This approach (asynchronous learning) enables introverted and anxious students learn in a safer and somewhat isolated environment. Asynchronous online learning environment has its demerits in that paucity of social interactions and delayed or absent feedback may work at cross purposes with the intended learning outcomes ( Oztok *et al*., 2013).

It will be prudent to use hybrid learning models which include a blend of both asynchronous and synchronous online learning which take advantage of both methods ( Yamagata-Lynch, 2014). The classes can be uploaded in advance and the discussion can be fixed after a time lapse. This provides time for students to reflect on what they are learning before answering questions or joining online discussions. It will allow learners to learn at their own pace, while still enabling a meaningful interaction through the discussions. However, a precaution needs to be exercised by instructors to assign a time frame for every unit. The flexibility offered to students in an e-learning format sometimes may act as the biggest deterrent to learning and results in inaction and procrastination. Assigning time frames to every unit and sharing a calendar indicating the timelines for each part is worthwhile. A built-in schedule planner should be integral to each component of the module to ensure the hybrid technique works well.

## Maxim 3: Tools

### Select the apt tool to have the maximum impact

Instructors are conversant with the course content but not always equally conversant in judging and selecting the ideal from the array of available e-learning tools.

Lauren Anstey and Gavan Watson ( Anstey and Watson, 2018) have suggested a user-friendly rubric which integrates a broad range of functional, technical, and pedagogical criteria for evaluation of online tools. The rubric’s evaluation criteria are divided into eight categories, against which the e-learning tool is evaluated. This could be used by instructors to zero in on the appropriate tool for the online interaction.

To exemplify, presently we need an e-learning tool that has flexibility in the timing, duration and class size. Moreover, it should be easy to use while offering guidance through user engagement; most of all, it must allow hypermobility which incorporates multiple forms of media (audio, video, and textual communication channel) to cater to different types of learners. An ideal tool must be cost effective, accessible through different platforms such as desktop, laptop, mobile phones and tablets. It should help learners integrate, rearrange, or extend new and existing information so that higher order thinking is explored. Most importantly if we want all learners to engage freely and effectively, we must establish a safe, trusting environment where personal information protection, data privacy and copy right issues need to be safeguarded ( Anstey and Watson, 2018). Ultimately, it is the instructor who will take the final call on which tool will work best for a particular course or group of students.

## Maxim 4: Trial run

### Try it! Test it! Tweak it!

The instructor needs to balance concern for the operation of the equipment with effective teaching. When technology is no longer viewed as a challenge, the instructor can focus more on the learning outcomes and innovative approaches ( Johnson *et al.*, 2016).

Once the training is completed, it is important for the online teacher to get well versed with the basic operation of the equipment like switching between sites, operating the switch buttons smoothly, manipulating cameras, controlling sound levels and using different options available with the technology at hand ( Simonson and Schlosser, 2014). The instructor must be acutely aware of the ability of the students to access the courseware through appropriate hardware and software. This has to be continuously seen from the viewpoint of the students.

In order to ensure all these, it is always wise to trial run the session prior to the original class. With the instructor’s familiarity with the technological tools, student learning becomes the bedrock on which every decision is taken. Establishing the ground rules and discussing them with students well in advance will ensure a smooth conduct of the online class.

## Maxim 5: Technical issues

### Not all students will have optimum access to gadgets

It is important to understand that some students may encounter challenges in accessing high speed internet consequent to which they may not be able to actively participate in the virtual classrooms. These technical issues make it hard for them to follow the online teaching, making their learning experience frustrating. Moreover, those who live in remote locations will find it difficult to meet the technical requirements of the chosen course ( Kumar, n.d.). The only solution to this problem is knowing exactly what kind of technological support they will need in advance so that the online activities can be planned. Offering multi-device online sessions where students can access the class via smartphones, browsers and operating systems gives students an opportunity to choose what suits them best. We should use online platforms that do not require much internal memory or a high-speed internet connection. We must keep it simple so that students do not have to download any program or print out documents. For example, converting important class files to PDFs can make it easier for students to use materials (Gamelearn Team, n.d.).

If the online tool which we have chosen has a chat service, email address or forum for sorting out technical glitches, it can be of great help and we can be certain that technology would not get in the way of the learning process.

## Maxim 6: Transition

### Be ready with plan B

Technical problems may arise even after meticulous planning of the online class. We need to be prepared for this. In case the synchronous session is interrupted, it is important for students to have projects and assignments independent of the instructor and alternative means of communication like e-mail, phone, etc. It is wise to discuss alternative strategies with students well in advance ( Simonson and Schlosser, 2014). Alternative lesson materials must always be ready although we hope it may not be needed. They must be designed to be used without instructor intervention. Recording of synchronous sessions is recommended so that it can be shared with the students just in case they lose connection midway.

## Maxim 7: Teacher preparation

### Teaching online differs from face- to- face sessions

In a traditional classroom, visual cues are extremely important to establish rapport with our learners. Visual cues from students personalizes learning and gives the instructor an idea regarding their current learning experience so that instructional modifications can be done ( Simonson and Schlosser, 2014).

In an online course it is more difficult to gather visual cues from and about students. In spite of using desktop conferencing technologies, it is not possible to visualize the entire class and their expressions on screen. If instructors ignore this area of preparation in online classes, there is huge risk that they may get frustrated as they have no clue whether the students are understanding what is being taught and also the students may get disconnected (pun intended) with the instructor.

To counteract this in an online communication, students need to be informed what is going to happen, what is expected of them and what they need to bring to the online class. Making the learning outcomes explicit will allow students to get accustomed to accept the online environment with ease.

Use of emoticons and infographics has been suggested to increase teaching presence in an online learning environment. Emojis can be used to encourage and appreciate when learners interact with each other, or with the online teacher ( Bai *et al*., 2019). They can be used to compliment students when they perform well in a task. However, too much of its usage during the discussion may hinder the learning process. Hence a balance needs to be achieved.

Another solution is to break the monotony by giving some tasks to students and involving them in the learning process which will give the teacher some assurance that students are alert and are on the same page with the instructor.

## Maxim 8: Teaching method

### Customize the teaching to suit online needs

There is as much diversity in teaching-learning strategies in online mode as in the face-to-face class encounters. The ideal method is that which engages the learner most. However, it is well established that the conventional lecture is the least effective method in the distance education mode ( Online Education, 2016).

Discussional method of learning works better than the didactic form especially in an online environment. Interactive training activities including simulations, videos, storytelling, gamified solutions, case studies, problem-based learning need to be used as teaching strategies to engage learners in the online environment ( Online Education, 2016). Dividing the class into subgroups of 10 to 15 students makes the online discussions more manageable, interesting and meaningful.

In synchronous sessions, instructors ask questions and discuss course material using real-time chats and web-conferencing tools. Whereas in asynchronous sessions students communicate via discussion boards, web forums and social media tools.

Another effective online technique to promote interaction in distance education is the threaded discussion where instructors post questions related to assignments and students post comments in a discussion area ( Rizopoulos and McCarthy, 2009).

## Maxim 9: Teamwork

### Allow student-student interaction

Instructors may try their best to make the online platform interesting and intriguing for the learners. In spite of their best efforts, the online environment can become too lonesome for the student. They get easily bored with online training, lose interest and complain of lack of engagement. This eventually leads to a failure of the e-learning platform. To prevent this boredom, we should make our classes interactive, collaborative, dynamic and fun-filled ( Paulus, 2005).

For the student, learning modules developed online should encompass personal responsibility, interaction, interdependence and creation of mutual understanding.

Most definitions of distance education revolve around Bates’s seventh golden rule that “interaction is essential” ( Simonson and Schlosser, 2014). Keegan noted that distance education must provide for two-way communication so that the student may not just interact but also initiate a discussion ( Sher, 2009).

Three forms of interaction are widely recognized in online learning ( Sher, 2009).

• student-content interaction
• student-instructor interaction
• student-student interaction

The emphasis today is on the third form embodying reflection, andragogy and constructivism.

Creating small groups and assigning them activities which can be shared with the entire class can be beneficial. Wikis work best for this ( Kear, Donelan and Williams, 2014).

Incentives like appreciation in the form of score boards reflecting their achievements, awarding stars for group tasks can keep up the team spirit and encourage students to participate more.

## Maxim 10: Testing

### Gauge student learning

It is important even in the online environment to ensure that the students are learning and achieving the learning outcomes. Online formative assessment strategies (through objective questions and assignments) built into the teaching learning activities will ascertain the depth of understanding ( Walsh, 2015).

Currently, online assessments are being criticized for lack of reliability due to which both learners and tutors lose their trust in assessments. The logistics of online assessment may engender lapses in the security which could give unfair advantage to those who exploit such deficiencies. Enough safeguards must ensure that this will not happen ( Walsh, 2015). However, when assessments are being done to foster learning, it matters little whether the assignments are done individually or severally.

## Conclusion

“Nobody can go back and start a new beginning, but anyone can start today and make a new beginning” - Maria Robinson

We summarize the ten maxims using a user-friendly checklist to guide online educators. ( Table 1)

**Table 1.** Checklist to prime online educators

Sr.no:	Items	If YES	If NO
1	Have you undergone training?	Proceed to next step	• Watch online tutorials/ webinars • Get in touch with your IT team • Some examples for tutorials • https://www.youtube.com/watch?v=9guqRELB4dg&vl=en - Zoom • https://www.youtube.com/watch?v=c_nWDZUb_bA WebEx • https://www.youtube.com/watch?v=DPZb3D0500I- Google hangout • https://www.youtube.com/watch?v=_Cw9uXfgTLQ- Microsoft Teams • https://www.youtube.com/watch?v=4Bpqla3Bxok- Blackboard Collaborate • https://www.youtube.com/watch?v=rCNImsWUxZA Google Classrooms
2	Did you decide on the technique of online learning you are going to use?	Proceed to next step	• Decide whether you prefer a synchronous or asynchronous technique. • We recommend a hybrid method (Upload your presentation on a common platform and send link to students to view. Fix a timing for discussion via a synchronous session)
3	Have you selected the appropriate tool?	Proceed to next step	• Select your tool now • Use Anstey and Watson’s Rubrics to help you choose • Some examples for asynchronous learning: Google classrooms, YouTube, WhatsApp, College websites like Moodle • Some examples for synchronous learning: Zoom, Hangout, Big blue button, MS teams, Skype, Blackboard collaborate, WebEx, any institution created platforms
4	Have you done a trial run of the session?	Proceed to next step	• Plan a trial run now • Send link to participants and inform them about the trial run • Try using all applications like sharing the screen, checking the video and audio buttons, checking the chat options, asking students to participate in interactive activities etc. • Note down the students’ feedback and reflect on your experience • Get in touch with your IT team if required
5	Have you taken care of technical issues which you anticipated /encountered during trial run?	Proceed to next step	• Check your internet connectivity • For example: Minimum bandwidth required for • Zoom meeting- 1.5Mbps • WebEx - 320Mbps • MS Teams - 1.2Mbps • Big Blue Button- 1Mbps • Prepare alternative if the Wi-Fi is not stable. Example: use mobile data • Select a multi-device tool if students want to access through mobile phones • Do not upload huge files which may require students to download • Share PDF documents which are easy to view on all devices • Consult your IT team
6	Are you ready for a transition to plan B if necessary?	Proceed to next step	• Plan projects and assignments independent of the instructor in case the synchronous session fails • Inform about this plan to students in advance • Record the session for later viewing as a backup
7	Are you aware of the different experience in online teaching?	Proceed to next step	• Be aware; visual cues may be missing • Try to interact with your students’ mid-way during the class with some activity, such as - asking to poll. • For example, real time polling sites like https://swift.excitem.com/ can be used to insert polling into your PowerPoint • Use emoticons for appreciating and encouraging students
8	Have you incorporated different methods of teaching in your online session?	Proceed to next step	• Be aware that a continuous lecture tends to bore the students • Incorporate case-based or problem-based discussions, simulations, educational games, storytelling and roleplays in your session • Use threaded discussions for a learner centered learning
9	Have you planned some group work for your students?	Proceed to next step	• Divide students into small groups and assign them tasks • Use wiki collaboration tool or Google forms Use this link to learn how to use a wiki https://www.youtube.com/watch?v=-dnL00TdmLY
10	Have you created formative assessments for students to respond to during and after the class?	Well done!	• Create assessments in the form of simple MCQ’s - You may use soft wares like Socrative https://www.youtube.com/watch?v=9hV2a6cxsic, https://b.socrative.com/login/teacher/ • Plan assignments which will enable teachers to gauge student understanding • Other soft wares like Gradescope have several options for assessments online and can be tried https://www.gradescope.com/get_started

In pandemic times like this everyone needs to do their bit to contribute to society. We need to outclass and outlast the academic challenges of COVID-19 pandemic by embracing the techniques of online teaching. As health professional teachers, we can adroitly ensure that learning is unfazed even by this biological cataclysm.

## Take Home Messages

• We should not let the COVID-19 pandemic adversely affect learning of health professions students.
• Faculty need to quickly and efficiently adapt themselves to the online platform.
• The checklist embodying the ten maxims can be used as a guide to equip faculty for this transition.

## Notes On Contributors

**Dr. Prashanti Eachempati** is working currently as Professor and Head of Prosthodontics at Faculty of Dentistry, Melaka Manipal Medical College, Malaysia. She is a FAIMER fellow (GSMC India), and the recipient of the prestigious Ron Harden Innovation in Medical Education Award in 2017. She has been a keynote speaker at various national and international conferences, continuing professional development programmes and conducted workshops at various institutions in medical/dental education related topics. ORCID iD: https://orcid.org/0000-0003-1263-7423.

**Dr K. Ramnarayan** was the fifth Vice-Chancellor of Manipal University from 2010 to 2015. He is currently the Vice President - Faculty Development, Manipal Academy of Higher Education, Manipal and Chancellor of Manipal University Jaipur. He is one of the early recipients of the ECFMG Foreign Faculty Fellowship in Basic Sciences. He was the recipient of the Bloomberg UTV Award for Outstanding Contribution to Education. He has more than 100 research publications, most of them pertaining to higher education. He has conducted more than 500 faculty development workshops in India and abroad. ORCID iD: https://orcid.org/0000-0002-7117-1830.

## Declarations

The author has declared that there are no conflicts of interest.

## Ethics Statement

Ethical approval was not required as this is a personal view/review article based on our experience.

## External Funding

This article has not had any External Funding
