# Supplementary material for: Ten maxims for out of class learning to outclass the academic challenges of COVID-19
Source: MedEdPublish (2016). 2020 May 5;9:89. [Version 1] doi: 10.15694/mep.2020.000089.1 (PMC10697550; doi:10.15694/mep.2020.000089.1)
Supplement: Peer review history

# Reviewer report: Reviewer response for version 1

**DOI:** 10.21956/mep.20047.r30709

**Published:** 2020-05-21

**Authors:** Raghavendra G

**Affiliations:** 1 Welcomgroup Graduate School of Hotel Administration

This review has been migrated. The reviewer awarded 5 stars out of 5 Apt and thoughtful for all the teachers on out of class learning.

Reviewer Expertise:

NA

No decision status is available

# Reviewer report: Reviewer response for version 1

**DOI:** 10.21956/mep.20047.r30708

**Published:** 2020-05-21

**Authors:** John Cookson

**Affiliations:** 1 University of Worcester

This review has been migrated. The reviewer awarded 5 stars out of 5 Thank you, yes agree with the other comments, a useful check list for anyone struggling with this; as most of us are.

Reviewer Expertise:

NA

No decision status is available

# Reviewer report: Reviewer response for version 1

**DOI:** 10.21956/mep.20047.r30716

**Published:** 2020-05-13

**Authors:** Puja Dulloo

**Affiliations:** 1 Pramukhswami Medical College

This review has been migrated. The reviewer awarded 4 stars out of 5 I really enjoyed reading this article. It has well highlighted tips for, out of class learning. In present pandemic state faculty members definitely are facing challenges not only in terms of adding digital innovations for their teaching learning and assessment aspect but coping their personal and family state. The biggest challenge is for the students who live in a village/ rural area where internet facilities are limited compared to those living in urban area. Inspite of a choice of online webinars sessions being arranged by various medical educationists in the country still many faculty members are facing difficulty, specifically, for assessment aspect. Hand-on training in must. Enthusiastic faculties do work for few of the portals at their own level, to understand it better and introduce it to the students for formative assessment part. This remembers me the challenge which my students encountered while working on their first formative assignment on Schoology app, although trial testing worked well for all the students. Few students could not complete their online assignment on time due to unstable network or difficulty to enter answer via a small keyboard in mobile, while few could not login to the app. So had to modify my assignment setting, like increased the number of attempts for completing the assignment, no grading system, increased the time duration to attempt for the assignment from 1 week to 3weeks. Students liked the prompt feedback for the assignment via this app and had liberty to ask for detailed explanation from the faculty directly.The ten tips really are required and would like to add few more like regular use of applications with the students, updating the knowledge by attending workshop session or courses.

Reviewer Expertise:

NA

No decision status is available

# Reviewer report: Reviewer response for version 1

**DOI:** 10.21956/mep.20047.r30713

**Published:** 2020-05-07

**Authors:** JAGAN MOHAN REDDY B R

**Affiliations:** 1 Dr.JAGANS SPECIALTY DENTAL CARE

This review has been migrated. The reviewer awarded 5 stars out of 5 A very well written article and is the need of the hour .Especially I like the checklist which gives a novice a systematic and easy way to shift from traditional to online platform . A very innovative presentation of the maxims using 10’T’s.Enjoyed Reading it.

Reviewer Expertise:

NA

No decision status is available

# Reviewer report: Reviewer response for version 1

**DOI:** 10.21956/mep.20047.r30715

**Published:** 2020-05-06

**Authors:** P Ravi Shankar

**Affiliations:** 1 American International Medical University

This review has been migrated. The reviewer awarded 4 stars out of 5 I enjoyed reading this paper about ten maxims for learning during the COVID-19 pandemic. The authors could use another term instead of ‘out of class’ in the title of the paper. In today’s world a class is no longer confined by physical boundaries and ‘blended’ learning strategies of on-site and remote sessions have been increasingly used even before the pandemic. Many medical schools especially in Australia and Canada used to offer sessions to their students who were posted in rural communities online. One of our assumptions is that students as digital natives will be well-placed to embrace and incorporate new technology. While this may be true to a certain extent there is still a learning curve for students and there will be a certain degree of getting used to virtual interaction and virtual learning. I believe training for the students is also important. Another point to note is that the tools will most often be adopted by the university or institution and while an individual instructor may have some freedom about which tool to use many might prefer to use institutionally supported technologies because of the benefits it offers. Bandwidth may be an issue for some students and home internet connections may not always offer the best bandwidth so asynchronous modes may sometimes be better with less emphasis on videos. The issues of online assessment may need to be elaborated on. Assessment in a purely virtual learning set up requires more thought. Maintaining student interest and keeping their spirits high during an online course is a challenge. This interesting article adds to the series of papers published in the journal on learning during the current pandemic.

Reviewer Expertise:

NA

No decision status is available

# Reviewer report: Reviewer response for version 1

**DOI:** 10.21956/mep.20047.r30711

**Published:** 2020-05-05

**Authors:** Felix Silwimba

**Affiliations:** 1 University of Lusaka

This review has been migrated. The reviewer awarded 4 stars out of 5 this is very informative document and provides practical guide to developing online education

Reviewer Expertise:

NA

No decision status is available

# Reviewer report: Reviewer response for version 1

**DOI:** 10.21956/mep.20047.r30712

**Published:** 2020-05-05

**Authors:** Subha Ramani

**Affiliations:** 1 Harvard Medical School, Brigham and Women's Hospital

This review has been migrated. The reviewer awarded 4 stars out of 5 The authors have provided a really useful list of maxims for all educators addressing the switch to virtual / online education. As they have stated, many of the teachers may not have experience in online formats and it would be wrong to assume that one just transposes in-person classes to an online environment. I believe they have covered key points for all teachers and educational designers to successfully navigate this transition.The article is written well, clear, easy to read and very useful to all educators during this period of change.I would add a couple of points that may not have been emphasized.1. Engaging students in co-construction of educational formats. Health professions education should start looking at students as collaborators and partners rather than recipients of education. After all, they are key stakeholders in education.2. Using IT expertise is good, we should also consider experts in educational technology. These experts may be hard to find in which case educationalists with technology training / expertise will be very useful additions to the curriculum planning team.

Reviewer Expertise:

NA

No decision status is available

# Reviewer report: Reviewer response for version 1

**DOI:** 10.21956/mep.20047.r30710

**Published:** 2020-05-05

**Authors:** Trevor Gibbs

**Affiliations:** 1 AMEE

This review has been migrated. The reviewer awarded 5 stars out of 5 A very well written, clear, concise and practical paper, from which I learned a lot.At a time like this during Covid-19, we not only need to be innovative but grounded; grounded in solid practical ways of development, in this case on-line learning.I suppose I would like to have seen a few more comments regarding challenges, especially from a resource-constrained country, but I feel that this paper is a must read for all those involved with curriculum design, development and delivery ( my d's do not match the authors' ingenuity with t's- sorry)

Reviewer Expertise:

NA

No decision status is available

# Reviewer report: Reviewer response for version 1

**DOI:** 10.21956/mep.20047.r30714

**Published:** 2020-05-05

**Authors:** Iriagbonse Osaigbovo

**Affiliations:** 1 N/A

This review has been migrated. The reviewer awarded 4 stars out of 5 I found this review apt for the times. A very well written and useful review.I think students' attitude and self-practice of online learning and their preferences should be assessed before hand to help plan this.

Reviewer Expertise:

NA

No decision status is available
